# Divergent presentation of anxiety in high-risk groups within the intellectual disability population

**DOI:** 10.1186/s11689-022-09462-w

**Published:** 2022-10-05

**Authors:** Laura Groves, Joanna Moss, Chris Oliver, Rachel Royston, Jane Waite, Hayley Crawford

**Affiliations:** 1 School of Psychology, University of Birmingham, Edgbaston, B15 2TT UK; 2 School of Psychology, University of Surrey, Guildford, GU2 7XH UK; 3 Division of Psychiatry, University College London, London, W1T 7NF UK; 4 School of Health and Life Sciences, Aston University, Birmingham, B4 7ET UK; 5 Mental Health and Wellbeing Unit, Warwick Medical School, University of Warwick, Coventry, CV4 7AL UK

**Keywords:** Anxiety, Genetic syndromes, Fragile X syndrome, Cornelia de Lange syndrome, Intellectual disability

## Abstract

**Background:**

Anxiety symptomatology is common in individuals with intellectual disability (ID). Symptomatology includes both traditional Diagnostic and Statistical Manual of Mental Disorders, 5th Edition (DSM-5) anxiety disorders and autism spectrum disorder (ASD)-related anxiety traits. Some genetic disorders such as Cornelia de Lange (CdLS) and fragile X syndromes (FXS) are at very high risk of anxiety and afford the opportunity to examine prevalence, profiles and associated person characteristics. However, prevalence and associated characteristics of anxiety in these high-risk groups remain poorly described and understood. The aim of the current study was to examine the prevalence and profile of DSM-5 and ASD-related anxiety symptomatology in individuals with CdLS and FXS and associated behavioural and cognitive characteristics.

**Methods:**

Questionnaires and interviews assessing DSM-5 and ASD-related anxiety were conducted with caregivers of individuals with CdLS (*n* = 49) and FXS (*n* = 36).

**Results:**

DSM-5 anxiety symptomatology was present in both groups with high co-morbidity across anxiety diagnoses. ASD-related anxiety was also prevalent with specific difficulties related to intolerance of uncertainty identified in both groups. Symptomatology was persistent over the lifespan for both groups. Anxiety type was partially associated with repetitive behaviour but not measures of overall ASD phenomenology in CdLS.

**Conclusions:**

DSM-5 and ASD-related anxiety are common in these high-risk syndromes associated with ID. Prospective syndrome specific presentations and associations, which may implicate specific underlying mechanisms, are discussed. Clinicians should be aware of the risk and difficulties involved in assessment of anxiety in individuals with ID, including atypical types, to ensure these individuals do not “miss” diagnoses and support in general clinical practice.

## Background

Anxiety is prevalent in individuals with intellectual disability (ID) and autistic individuals (approximately 27–50% [1, 2]) with high comorbidity between anxiety types [3]. Cornelia de Lange syndrome (CdLS) and fragile X syndrome (FXS) are two genetic syndromes associated with ID and autism spectrum disorder (ASD), and both reported to be at high risk of showing behaviours indicative of anxiety [4–8]. CdLS is a cohesinopathy associated with ID (estimated prevalence 1:10,000–1:30,000) [9]. FXS results from the silencing of the FMR1 gene leading to absence of the fragile X messenger ribonucleoprotein 1^2^ [10, 11] (estimated prevalence 1:4,000 males and 1:8,000 females) [10, 11].

Symptomatology indicative of anxiety is present in up to 64% of individuals with CdLS [5, 7–9]. Typically, this is categorised as social anxiety-like with high rates of selective mutism reported [12–14]. However, more recent evidence indicates that people with CdLS may show symptomatology suggestive of a broader anxiety profile, including generalised anxiety, separation anxiety, panic disorder and agoraphobia [15]. Similar to individuals with CdLS, high rates of anxiety disorder symptomatology are reported in FXS with approximately 70–86.2% of individuals meeting criteria for at least one anxiety disorder [4, 15]. In the first study to examine the prevalence of anxiety disorders against DSM-IV criteria, the most common forms of anxiety were specific phobias, social anxiety and selective mutism [16]. Other evidence suggests generalised anxiety and obsessive compulsive disorder may also be relatively common disorders in this group (approximate prevalence of 27% each [4, 15]). Despite evidence of anxiety symptomatology in individuals with CdLS and FXS, the profiles and prevalence rates of these remains poorly understood.

Diagnoses of anxiety disorders in individuals with ID is challenging due to diagnostic overshadowing, impaired cognitive and expressive language skills, difficulties identifying emotions and possible atypical presentations [12, 13]. For example, in individuals with FXS, when diagnostic criteria requiring people to self-report on internal thought processes were excluded, the rates of social anxiety increased from 34.5 to 60.3% [6]. Consequently, many individuals may not be considered to meet full criteria for diagnosis but could be described as showing subthreshold symptomatology for Diagnostic and Statistical Manual of Mental Disorders, 5th Edition (DSM-5) disorders [17], which may be of clinical significance. Examining profiles of DSM-5 symptomatology at *subthreshold* level in individuals with ID is critical as these are likely to include cases of missed threshold diagnoses. Moreover, individuals with subthreshold conditions experience significant deleterious outcomes to their wellbeing and quality of life [18] and consequently may represent a group of individuals with significant unmet need. There is also emerging evidence that other anxiety types may be present in autistic individuals [3]. These *ASD-related* anxiety types are hypothesised to occur downstream from sensory hyperarousal and intolerance to uncertainty [16, 19]. Whilst not captured within clinical guidelines, ASD-related anxiety has a detrimental impact on wellbeing and quality of life and is thus a critical area when assessing anxiety in individuals with ID where ASD characteristics are evident [12, 16, 19]. Individuals with CdLS and FXS are reported to show high rates of ASD characteristics with 43% and 30% of individuals estimated to meet criteria for diagnosis [20]. Despite the high prevalence of autistic characteristics reported for these groups of people, no studies to our knowledge have explored symptomatology for ASD-related anxiety in CdLS and FXS.

Accurate assessment of anxiety symptomatology in individuals who also show autistic characteristics is challenging due to the potential for overlap between these constructs (e.g. social withdrawal) [4]. This is important to consider when assessing anxiety in syndromes such as CdLS and FXS where behaviour indicative of social anxiety is commonly reported alongside difficulties with of social interactions consistent with autism [12, 13]. Cross-syndrome comparisons of groups with known genetic aetiologies who are at high risk for anxiety disorders allow syndrome related profiles to be identified, whilst controlling for confound variables such as level of ID and ASD phenomenology [21]. Additionally, where the genetic aetiology is known, underlying biological mechanisms of presentations may be evaluated. Critically, these syndromes make useful contrast groups as they are broadly comparable for level of ability and presence of ASD characteristics [22]. Person characteristics associated with anxiety symptomatology should also be examined to identify putative risk markers and prospective causal pathways. Specifically, greater chronological age, level of ability, severity of ASD characteristics and presence of repetitive behaviours may be associated with anxiety symptomatology [4, 12, 18, 23, 24].

In summary, whilst anxiety symptomatology is considered prevalent in both syndromes, presentation, profile and associated person characteristics remain poorly understood. Additionally, previous research has indicated that the presentation of anxiety in CdLS and FXS may be atypical both with regard to the specific nature and behavioural presentation of anxiety [7, 12]. However, in order to better understand the presentation of anxiety to inform clinical practice, it is important to apply existing frameworks of anxiety to evaluate how these may effectively characterise anxiety in these syndrome groups before atypical forms of anxiety are explored. Thus, examination of subthreshold DSM-5 and ASD-related anxiety is a critical first step in characterising and understanding anxiety in people with genetic syndromes, as well as people with ID in general. No study to date has investigated symptomatology against DSM-5 classifications or ASD-related anxiety in CdLS. This is particularly important given literature highlighting that people with these presentations experience poorer quality of life and wellbeing and may represent a group of individuals with unmet need.

In this study, we evaluate profiles of anxiety in two genetic syndrome groups associated with ID, reported to be at increased risk for anxiety. Group selection and the general approach enables cross-syndrome comparisons to be made whilst controlling for chronological age, level of ID and presence of ASD characteristics. Where syndrome-associated differences in the presentation of anxiety are identified, underlying biological or cognitive mechanisms may be implicated. The primary aim is to assess the presence and comorbidity of anxiety symptomatology in these syndrome groups. A secondary aim is to establish whether person characteristics including chronological age, adaptive functioning, ASD characteristics and repetitive behaviour are associated with anxiety symptomatology.

## Methods

### Participants

Caregivers of individuals with CdLS (*n* = 49) and FXS (*n* = 36) were recruited via a database held at the Cerebra Network for Neurodevelopmental Disorders at the University of Birmingham, the Cornelia de Lange Syndrome Foundation, UK and Ireland, or the Fragile X Society, UK. Participants were included if the person they cared for was mobile, at least 3 years old and had a confirmed genetic or clinical diagnosis of CdLS or a genetic diagnosis of FXS. Demographic information is shown in Table 1. No group significant differences for chronological age, adaptive functioning or ASD characteristics were found (all *p* > .05). As anticipated, there was a significant group difference in gender because only males with FXS were recruited for this study due to phenotypic gender differences [21].

**Table 1:** Demographic information for participants with Cornelia de Lange and fragile X syndromes

		CdLS	FXS	***t***/***U***/***χ***^**2**^	***df***	***p*** value
*n*		49	36			
Age (years)	Median (interquartile range)	15.92 (9.89–29.79)	20.63 (13.81–20.63)	754.50		.26
Gender	% Male	42.90	100.00	30.67	1	**< .001**
Speech	% Verbal	77.55	100.00	--^a^		**.002**
VABS-II Adaptive Behavior Composite (SS)	Median (interquartile range)	53.00 (25.00–68.00)	53.00 (41.25–53.00)	843.50		.73
SRS-2 total *t*-score	Mean (*SD*) Range	71.02 *(10.42)* 47–90	73.26 (8.52) 50–90	− 1.030	79	.31
Scoring above cut-off for autism^b^	*n (%)*	29 (59.18)	18 (36.73)	--^a^		.721

^a^Where Fishers exact tests were employed only *p*-values could be reported

^b^As indicated by the SRS-2

*Note. n* may vary due to missing data. *VABS-II* Vineland Adaptive Behavior Scale—Second Edition [22], *SRS-2* Social Responsiveness Scale—2 [25]

### Measures

#### Vineland Adaptive Behavior Scale

The Vineland Adaptive Behavior Scale-Second Edition (VABS-II) [26] is an informant interview assessing adaptive behaviour across four domains: communication, daily living, socialisation and motor skills. From these, an Adaptive Behavior Composite (ABC) standard score may be derived. Internal consistency and convergent validity are reported to be high [27]. The VABS-II ABC was used to determine whether participant groups were broadly comparable for overall adaptive functioning and to analyse associations with measures of anxiety.

#### The Social Responsiveness Scale

The Social Responsiveness Scale-Second Edition (SRS-2) [28] is an informant questionnaire assessing severity of ASD characteristics across five subscales: social awareness, social cognition, social communication, social motivation and restricted interests and repetitive behaviour. *T*-scores may be calculated with higher scores indicating greater deficit. *T*-scores of ≥ 60 are considered indicative of ASD and ≥ 75 indicative of autism. The SRS-2 is reported to have excellent internal consistency [28] and good specificity and sensitivity [22, 29]. Additionally, internal consistency calculated for the current sample was excellent (Cronbach’s alpha: CdLS = .928; FXS = .902). The SRS-2 total t-scores was used to ensure groups were comparable for severity of ASD symptomatology and to analyse associations with measures of anxiety.

#### Repetitive Behaviour Questionnaire

The Repetitive Behaviour Questionnaire (RBQ) [30] is an informant questionnaire assessing presence of repetitive behaviour in individuals with ID. There are five subscales: stereotyped behaviour (3 items, score range = 0-12), compulsive behaviour (8 items, score range = 0–24), restricted preferences (3 items, score range = 0–12), repetitive speech (3 items, score range = 0–12) and insistence on sameness (2 items, score range = 0–8). The RBQ is reported to have good internal consistency, content validity, concurrent validity, test–retest reliability and inter-rater reliability [30]. Internal consistency calculated for the current sample was excellent (Cronbach’s alpha: CdLS = .896; FXS = .831). Higher scores on the RBQ indicate greater frequencies of repetitive behaviour. The RBQ was used to analyse associations between each subscale and measures of anxiety.

#### Anxiety Scale for Children-Autism Spectrum Disorder

The Anxiety Scale for Children-Autism Spectrum Disorder parent version (ASC-ASD) [31] is an informant questionnaire used to measure anxiety-related items relevant to the specific phenomenology of anxiety in ASD. The ASC-ASD has four subscales: performance anxiety (5 items, score range = 0–15), uncertainty (8 items, score range = 0–24), anxious arousal (6 items, score range = 0–18) and separation anxiety (5 items, score range = 0–15). A composite total score may be calculated (score range = 0–72) with scores of **≥** 20 indicative of significant levels of anxiety. The ASC-ASD is reported to have good to excellent internal consistency, test-retest reliability and convergent validity [31]. Additionally, internal consistency calculated on the current sample was identified as good to excellent (Cronbach’s alpha: CdLS = .928; FXS = .742). Subscale and total scores from the ASC-ASD was used to establish prevalence of ASD-related anxiety types and associations with participant characteristics.

#### Kiddie-Schedule of Affective Disorders and Schizophrenia

The Kiddie-Schedule of Affective Disorders (K-SADS) [32] is a semi-structured informant neuropsychiatric interview based on the DSM-5 [17]. The K-SADS is reported to have robust psychometric properties and high test-retest reliability [33] and has been used with children and adults with ID previously [34, 35]. The K-SADS was used to determine current presence of symptomatology consistent with the following anxiety types: panic, agoraphobia, separation anxiety, social anxiety, selective mutism, specific phobias, generalised anxiety, obsessive compulsive, trauma and stressor-related disorders. The K-SADS was not used as a full diagnostic tool due to the difficulties of diagnosis in individuals with severe to profound ID [12, 36]. Instead, the purpose was to determine whether behaviours indicative of each type of anxiety were shown by individuals at subthreshold level (scored as present or absent) as determined by the screening interview. Individuals flagged as showing presence of subthreshold symptomatology suggest the person is showing behaviour indicative of that anxiety disorder; however, a full assessment of criteria (e.g. of self-reported items) was not undertaken. Inter-rater reliability conducted on 10 interviews was good (kappa = .695; percentage agreement = 89%). A DSM-5 Anxiety Composite was computed by tallying the number of subthreshold DSM-5 anxiety types an individual was reported to show symptomatology for (maximum score = 9).

### Procedure

Once written informed consent was received, caregivers were sent a questionnaire pack and interviews were conducted over the telephone.

### Data-analysis

Distribution of data was assessed using Kolmogorov-Smirnov tests, visual inspection of QQ plots and examination of skewness and kurtosis. For non-normally distributed data, and where methods of transformation were unsuccessful, non-parametric alternatives were employed. Multiple comparisons were being made; however, as this was an exploratory analysis, an alpha level of *p* < .05 was used throughout [37]. Analyses are not reported where group membership was *n* < 10 as this was considered insufficient for analysis.

#### DSM-5 and ASD-related symptomatology presence and co-occurrence

To address the primary aim of this study, the presence and co-occurrence of anxiety in CdLS and FXS, presence of DSM-5 and ASD-related anxiety were explored. Additionally, co-occurrence of DSM-5 anxiety types and associations between DSM-5 anxiety and ASD-related anxiety were analysed.

Group differences in prevalence rates for DSM-5 anxiety symptomatology, chi-square and Fisher’s exact tests were conducted on presence of subthreshold DSM-5 disorder symptomatology, as measured by the K-SADS. To assess rates of co-occurrence, associations between the presence of one disorder with a second disorder was assessed using chi-square and Fisher’s exact tests within each syndrome group. To explore profiles of ASD-related anxiety, group comparisons of ASC-ASD subscales and total scores were conducted using *t*-tests. Group differences in the number of individuals meeting cut-off levels for significant anxiety [31] were assessed using chi-square and Fisher’s exact tests. Due to the level of expressive communication required by the ASC-ASD, minimally verbal individuals^1^ were excluded in this analysis (included participants: CdLS = 36; FXS = 31). Additionally, to investigate how ASD-related anxiety was associated with the presentation of DSM-5 subthreshold anxiety types in these groups, 2 (DSM-5 anxiety: present, absent) x 2 (syndrome: CdLS, FXS) ANOVAs were conducted on ASC-ASD subscale scores. Due to assumption violations, these could only be conducted for scores on generalised anxiety, specific phobias as indicated by the K-SADS and the uncertainty subscale of the ASC-ASD.

#### Associated person characteristics

The second aim of this study was to establish whether person characteristics, including chronological age, adaptive functioning, ASD characteristics and repetitive behaviour, were associated with anxiety symptomatology.

Within syndrome group analyses were conducted between individual anxiety types and participant characteristics: chronological age, adaptive functioning, ASD symptomatology and repetitive behaviour. Due to sample sizes, these analyses were only conducted for the five most prevalent DSM-5 anxiety types reported here which were specific phobias, social anxiety, agoraphobia, generalised anxiety and separation anxiety. Additionally, only the uncertainty subscale of the ASC-ASD was used as this was the most highly endorsed ASD-related anxiety type in both groups and the only subscale with spread of data determined sufficient for analysis. For K-SADS data, the CdLS and FXS groups were subdivided into groups where symptomatology was present at subthreshold or absent (CdLS-anxiety, CdLS-none, FXS-anxiety, FXS-none). Chi-square tests were employed to investigate associations with presence of DSM-5 symptomatology. Chronological age bands (< 18 and ≥ 18 years) were selected so the older group best represented an adult population. Adaptive behaviour bands (VABS-II ABC standard score: < 50 and ≥ 50) were selected based on a mean-split of the data. Finally, bands for ASD characteristic (SRS-2 *t*-score: < 75 and ≥ 75) were selected as a *t*-score of 75 is considered indicative of autism. Group differences for DSM-5 anxiety types (present at subthreshold versus absent) on RBQ subscales were conducted using *t*-tests and to establish associations with repetitive behaviour. As uncertainty subscale scores were continuous, Spearman’s correlations were employed to establish associations with chronological age, VABS ABC standard scores, total SRS *t*-scores and the RBQ subscales.

## Results

### DSM-5 symptomatology

The prevalence of anxiety symptomatology was strikingly high in both groups. Overall, 91.8% and 100.0% of individuals with CdLS and FXS, respectively, were reported to show symptomatology for a minimum of one anxiety disorder. Symptomatology consistent with a minimum of two anxiety types was evident in 81.6% and 88.9% of individuals with CdLS and FXS respectively. The prevalence of subthreshold DSM-5 anxiety symptomatology for the CdLS and FXS groups are reported in Table 2. Social anxiety was more prevalent in the FXS group compared to the CdLS group (CdLS = 53.1%; FXS = 75.0%; *χ*^2^(1) = 4.26, *p* < .05; ϕ_c_ = .224). No other significant group differences were identified. However, the *p*-value for selective mutism approached significance such that this appeared more prevalent in the CdLS group (CdLS = 32.5%; FXS = 13.8%; *χ*^2^(1) = 3.63, *p* = .057 ϕ_c_ = .207).

**Table 2:** Group comparisons of prevalence of DSM-5 symptomatology in individuals with Cornelia de Lange and fragile X syndromes

	CdLS ***n*** (%)		FXS ***n*** (%)		***χ***^**2**^	***p***-value	Cramer’s ***V***
	Present	Not present	Present	Not present			
Panic	6 (17.1)	43 (87.8)	1 (2.8)	35 (97.2)	--^a^	.230	-- ^b^
Agoraphobia	25 (51.0)	24 (49.0)	25 (69.4)	11 (30.6)	2.908	.088	.185
Separation anxiety	17 (35.4)	31 (64.6)	10 (27.8)	26 (72.2)	.754	.385	.094
Social anxiety	26 (53.1)	23 (46.9)	27 (75.0)	9 (25.0)	4.255	**.039**	.224
Selective mutism	13 (32.5)	27 (67.5)	5 (13.9)	31 (86.1)	3.631	.057	.207
Specific phobias	37 (75.5)	12 (24.5)	25 (69.4)	11 (30.6)	.387	.534	.067
Generalised anxiety	22 (44.9)	27 (55.1)	20 (55.6)	16 (44.4)	.943	.332	.105
Obsessive compulsive	13 (26.5)	36 (73.5)	5 (13.8)	31 (86.1)	1.987	.159	.153
Trauma and stressor-related disorders	8 (16.3)	41 (83.7)	4 (11.4)	32 (88.9)	--^a^	.547	-- ^b^

^a^Where Fishers exact tests were employed only *p*-values could be reported

^b^Cramer’s *V* could only be calculated for chi-square analyses

To assess rates of co-occurrence, associations between the presence of one disorder with a second disorder was assessed within each syndrome group (see Table 3). In the CdLS group, social anxiety symptomatology was significantly associated with selective mutism (*χ*^2^(1) = 14.235, *p* < .001, ϕ_c_ = .60). Agoraphobia was associated with social anxiety (*χ*^2^(1) = 10.783, *p* < .005, ϕ_c_ = .47) and generalised anxiety symptomatology (*χ*^2^(1) = 15.154, *p* < .001, ϕ_c_ = .56). Both the CdLS and FXS groups showed associations between separation anxiety and specific phobias (both groups: *p* < .05). Finally, the FXS group showed significant associations between separation anxiety and obsessive-compulsive symptomatology (*p* < .05). No other associations in the CdLS or FXS groups were observed (*p* > .05).

**Table 3:** Within group associations between DSM-5 anxiety in Cornelia de Lange and fragile X syndromes indicating co-morbidity

		Agoraphobia	Separation anxiety	Social anxiety	Selective mutism	Specific phobias	Generalised anxiety	Obsessive compulsive	Trauma and stressor-related disorders
**CdLS**		*n* = 25	*n* = 17	*n* = 26	*n* = 13	*n* = 37	*n* = 22	*n* = 13	*n* = 8
*n* (%) also scoring for:	*Agoraphobia*	--	11 (64.7)	19 (73.1)**	10 (76.9)	22 (59.5)	18 (81.8)***	9 (69.2)	2 (25.0)
	*Separation anxiety*	11 (44.0)	--	11 (42.3)	3 (23.1)	17 (45.9)*	7 (31.8)	6 (46.2)	1 (12.5)
	*Social anxiety*	19 (76.0)**	11 (64.7)	--	13 (100.0)***	22 (59.5)	15 (68.2)	10 (76.9)	3 (37.5)
	*Selective mutism*	10 (40.0)	3 (17.6)	13 (50.0)***	--	12 (32.4)	8 (36.4)	5 (38.5)	2 (25.0)
	*Specific phobias*	22 (88.0)	17 (100.0)*	22 (84.6)	12 (92.3)	--	19 (86.4)	13 (100)	5 (62.5)
	*Generalised anxiety*	18 (72.0)***	7 (41.2)	15 (57.7)	8 (61.5)	19 (51.4)	--	8 (61.5)	2 (25.0)
	*Obsessive compulsive*	9 (69.2)	6 (35.3)	10 (38.5)	5 (38.5)	13 (64.9)	8 (36.4)	--	2 (25.0)
	*Trauma and stressor-related disorders*	2 (8.0)	1 (5.9)	3 (11.5)	2 (15.4)	5 (13.5)	2 (9.1)	2 (15.4)	--
**FXS**		*n* = 25	*n* = 10	*n* = 27	*n* = 5	*n* = 25	*n* = 20	*n* = 5	*n* = 4
*n* (%) also scoring for:	*Agoraphobia*	--	9 (90.0)	21 (77.8)	5 (100.0)	17 (68.0)	15 (75.0)	5 (100.0)	3 (75.0)
	*Separation anxiety*	9 (36.0)	--	8 (29.6)	2 (40.0)	10 (40.0)*	7 (35.0)	4 (80.0)*	3 (75.0)
	*Social anxiety*	21 (84.0)	8 (80.0)	--	5 (100.0)	17 (68.0)	17 (85.0)	5 (100.0)	3 (75.0)
	*Selective mutism*	5 (20.0)	2 (20.0)	5 (18.5)	--	2 (8.0)	3 (15.0)	2 (40.0)	1 (25.0)
	*Specific phobias*	17 (68.0)	10 (100.0)*	17 (63.0)	2 (40.0)	--	15 (75.0)	5 (100.0)	3 (75.0)
	*Generalised anxiety*	15 (60.0)	7 (70.0)	17 (63.0)	3 (30.0)	15 (60.0)	--	3(60.0)	2 (50.0)
	*Obsessive compulsive*	5 (20.0)	4 (40.0)*	5 (18.5)	2 (40.0)	5 (20.0)	3 (15.0)	--	2 (50.0)
	*Trauma and stressor-related disorders*	3 (12.0)	3 (30.0)	3(11.1)	1 (20.0)	3 (12.0)	2 (10.0)	2 (40.0)	--

*p < 0.05; **p < 0.01; ***p < 0.001

### ASD-related anxiety

Cross-syndrome comparisons of ASD-related anxiety types showed no significant differences for subscale and total scores using the ASC-ASD (*p* > .05; see Table 4). Fourteen (43.8%) participants with CdLS and 11 (35.5%) participants with FXS scored above the proposed cut off for total scores. Only the uncertainty subscale met assumptions for the ANOVA analysis and so two, 2 (anxiety presence) x 2 (syndrome) mixed ANOVAs with uncertainty as the independent variable were computed for specific phobias and generalised anxiety. No main effects were shown for specific phobias (*F*(3, 66) = .962, *p* > .05). However, significant differences were shown in the generalised anxiety analysis (*F*(3, 66) = 4.1762, *p* < .01). This was driven by a main effect of presence of anxiety such that individuals with generalised anxiety showed higher scores for the uncertainty subscale (absent: mean = 7.85, *SD* = *5.14*; present: mean = 12.14, *SD* = 5.33; *F*(1, 66) = 12.183, *p* < .01). No interaction effects were identified (*p* > .05).

**Table 4:** Syndrome group comparisons on the ASC-ASD for verbal participants only

		CdLS (*n* = 36)	FXS (*n* = 31)	***t***/***U***/***χ***^**2**^	***df***	***p*** value	Effect size^**a**^
Performance anxiety	Median (interquartile range)	2.00 (0.00–5.00)	0.00 (0.00–4.25)	455.50		.255	.140
Arousal anxiety		1.00 (0.00–3.00)	1.00 (0.00–2.00)	395.00		.077	.219
Separation anxiety		3.00 (1.00–4.00)	1.00 (0.00–4.00)	455.00		.265	.137
Uncertainty^b^		9.50 (6.00–11.75)	11.00 (5.00–15.00)	− .508	65	.613	.120
Total score^b^		16.00 (9.13–22.94)	15.57 (9.00–23.00)	.543	65	.589	.190
Meeting cut-off^c^	*n* (%)	14 (43.75)	11 (35.48)	.083	1	.805	.036

^a^Cohen’s *d* reported for *t*-tests, *r* for Mann-Whitney *U* tests, Cramer’s *V* for chi-square tests

^b^Uncertainty and total score subscales calculated using a square root transformation

^c^Cut-off indicating significant anxiety [27]

### Associated characteristics

Within syndrome group comparisons were conducted between chronological age, adaptive behaviour, ASD symptomatology and repetitive behaviour for uncertainty and the five most prevalent DSM-5 anxiety types reported by both syndrome groups: agoraphobia, separation anxiety, social anxiety, specific phobias and generalised anxiety. To aid interpretation, the person characteristics of the subgroups created for these analyses are presented in Table 5.

**Table 5:** Characteristics of CdLS and FXS subgroups used for within syndrome analyses of prevalence of anxiety symptomatology and chronological age, level of ability and severity of ASD symptomatology

		Chronological age (years)		VABS-II ABC standard score (SS)		SRS-2 total ***t***-scores	
		***< 18 years***	***≥ 18 years***	< 50	≥ 50	< 75	≥ 75
**CdLS**	***n*** **(%)**	25 (51.02)	24 (48.98)	23 (46.94)	26 (53.06)	29 (59.18)	18 (36.73)
	**Mean** ***(SD)*, range**	9.48 *(3.68)*, 3.42–15.92	31.32 *(8.22)*, 18.08–53.50	30.61 *(11.22)*, 20.00–49.00	65.54 *(7.02)*, 50.00–79.00	64.31 *(6.12)*, 47.00–73.00	81.83 *(5.60)*, 75.00–90.00
**FXS**	***n*** **(%)**	14 (38.89)	22 (61.11)	16 (44.44)	20 (55.56)	18 (50.00)	16 (44.44)
	**Mean** ***(SD)*, range**	11.69 *(3.65)*, 6.50–17.92	29.63 *(8.86)*, 18.83–48.42	39.13 *(7.46)*, 20.00–49.00	62.95 *10.62)*, 52.00–93.00	66.83 *(5.38)*, 50.00–73.00	80.50 *(4.63)*, 75.00–90.00

Table 6 presents the within syndrome group comparisons for chronological age, adaptive behaviour and ASD characteristics. For chronological age, only the FXS group showed significant associations between age and the presence of specific phobias, such that the younger group were more likely to show evidence of specific phobias symptomatology (< 18 years = 92.9% and ≥ 18 years = 54.5%, *p* < .05; see Table 6). For adaptive behaviour, only specific phobias in the CdLS group were associated with VABS-II ABC standard scores such that specific phobias were reported more in the lower ability group (< 50 = 55.6% and ≥ 50 = 44.4%, *χ*^2^(1) = 5.483, *p* < .05, ϕ_c_ = .34). For ASD characteristics, only uncertainty was associated with SRS-2 *t*-scores in both the CdLS and FXS groups (CdLS: *r* = .401, *p* = .006; FXS: *r* = .464, *p* = .009). No other significant associations were reported (*p* > .05).

**Table 6:** Within syndrome associations of prevalence of anxiety symptomatology with chronological age, level of ability and severity of ASD symptomatology

	CdLS					FXS				
**Chronological age**	***n*** **(%)**		***χ***^**2**^	***p*-value**	**Cramer’s** ***V***	***n*** **(%)**		***χ***^**2**^	***p*-value**	**Cramer’s** ***V***
	**< 18 years**	**≥18 years**				**< 18 years**	**≥18 years**			
*Agoraphobia*	13 (50.00)	12 (52.17)	.023	1.000	.022	11 (78.57)	14 (63.64)	--^a^	.467	--^b^
*Separation anxiety*	10 (40.00)	7 (30.43)	.479	.556	.100	5 (35.71)	5 (22.73)	--^a^	.318	--^b^
*Social anxiety*	12 (46.15)	14 (60.87)	1.061	.229	.147	11 (78.57)	16 (72.73)	--^a^	.506	--^b^
*Specific phobias*	19 (73.08)	18 (78.26)	.177	.466	.060	13 (92.86)	12 (54.55)	--^a^	.025*	--^b^
*Generalised anxiety*	9 (34.62)	13 (56.52)	2.367	.105	.220	10 (71.43)	10 (45.45)	2.338	.176	.255
**VABS-II ABC standard score**	***n*** **(%)**		***χ***^**2**^	***p*-value**	**Cramer’s** ***V***	***n*** **(%)**		***χ***^**2**^	***p*-value**	**Cramer’s** ***V***
	**< 50**	**≥ 50**				**< 50**	**≥ 50**			
*Agoraphobia*	14 (58.33)	10 (41.67)	3.021	.147	.251	11 (44.00)	14 (56.00)	--^a^	1.000	--^b^
*Separation anxiety*	9 (56.25)	7 (43.75)	.869	.376	.136	5 (50.00)	5 (50.00)	--^a^	.722	--^b^
*Social anxiety*	14 (56.00)	11 (44.00)	2.172	.161	.213	12 (44.44)	15 (55.56)	--^a^	1.000	--^b^
*Specific phobias*	20 (55.56)	16 (44.44)	5.483	.042*	.338	10 (40.00)	15 (60.00)	--^a^	.483	--^b^
*Generalised anxiety*	12 (57.14)	9 (42.86)	1.923	.244	.200	8 (40.00)	12 (60.00)	.350	.737	.100
**SRS-2 total** ***t*-scores**	***n*** **(%)**		***χ***^**2**^	***p*-value**	**Cramer’s** ***V***	***n*** **(%)**		***χ***^**2**^	***p*-value**	**Cramer’s** ***V***
	**< 75**	**≥ 75**				**< 75**	**≥ 75**			
*Agoraphobia*	13 (44.83)	10 (55.56)	.512	.556	.104	12 (66.67)	12 (66.67)	--^a^	.715	--^b^
*Separation anxiety*	7 (25.00)	8 (44.44)	1.885	.208	.202	3 (16.67)	6 (37.50)	--^a^	.250	--^b^
*Social anxiety*	15 (51.72)	10 (55.56)	.065	1.000	.037	13 (72.22)	12 (75.00)	--^a^	1.000	--^b^
*Specific phobias*	20 (68.97)	15 (83.33)	--^a^	.324	--^b^	12 (66.67)	11 (68.75)	.017	1.000	.022
*Generalised anxiety*	13 (44.83)	8 (44.44)	.001	1.000	.005	10 (55.56)	9 (56.25)	.002	1.000	.008

*p < .05

*Note. n* may vary due to missing data

^a^Where Fishers exact tests were employed only *p*-values could be reported

^b^Cramer’s *V* could only be calculated for chi-square analyses

Comparisons were conducted between repetitive behaviour and DSM-5 anxiety for the CdLS group only as the *n* values for the FXS group were insufficient for analyses (*n* < 10). These are presented in Table 7 and show agoraphobia was associated with restricted preferences (*U* = 99.50, *p* < .05, *r* = .181), separation anxiety was associated with compulsive behaviour (*t*(44) = − 2.650, *p* < .05, *d* = .777) and specific phobias was associated with repetitive language (*U* = 63.50, *p* < .05, *r* = .238) such that presence of symptomatology was associated with greater frequency of repetitive behaviour. Finally, data for the uncertainty subscale was shown to be correlated with restricted preferences (*r*(33) = .350, *p* < .05) and insistence on sameness (*r*(33) = .360, *p* < .05). No other significant associations were found (*p* < .05)

**Table 7:** Group comparisons of repetitive behaviour between individuals with Cornelia de Lange syndrome reported to show presence or absence of anxiety symptomatology

	Median (interquartile range)		***t***/***U***	df	***p***-value	Effect size^**a**^
	Symptomatology present	Symptomatology not present				
**Stereotyped behaviour**						
*Agoraphobia*	4.00 (0.00–8.00)	3.50 (0.00–6.00)	241.00		.449	.110
*Separation anxiety*	7.00 (3.00–11.00)	3.00 (0.00–6.00)	157.50		.053	.347
*Social anxiety*	2.00 (0.00–8.00)	4.00 (1.50–7.25)	242.50		.481	.103
*Specific phobias*	4.00 (0.00–8.00)	3.50 (0.00–4.00)	171.50		.340	.139
*Generalised anxiety*	2.00 (0.00–7.50)	4.00 (1.50–8.00)	234.00		.396	.124
**Compulsive behaviour**						
*Agoraphobia*	7.00 (2.00–13.00)	5.00 (.00–11.50)	− 1.162	45	.251	.339
*Separation anxiety*	11.00 (6.00–19.00)	4.00 (.00–12.00)	− 2.650	44	.011*	.777
*Social anxiety*	5.00 (1.50–12.50)	10.00 (.75–13.00)	.232	45	.818	.069
*Specific phobias*	7.00 (2.00–13.00)	4.50 (0.00–12.50)	− .892	45	.377	.311
*Generalised anxiety*	8.00 (1.50–13.00)	4.50 (0.75–11.25)	227.00		.322	.144
**Restricted preferences**						
*Agoraphobia*	5.00 (3.75–10.25)	4.00 (1.00–7.00)	99.50		.034*	.181
*Separation anxiety*	5.00 (4.00–10.50)	5.00 (3.00–7.00)	− 1.481	34	.148	.541
*Social anxiety*	5.00 (1.50–12.50)	6.00 (1.50–9.50)	154.50		.841	.117
*Specific phobias*	5.00 (3.00–7.50)	4.00 (1.50–10.75)	− .895	45	.377	.332
*Generalised anxiety*	5.00 (3.00–10.50)	4.00 (3.25–6.75)	− 1.281	33.41	.195	.429
**Repetitive language**						
*Agoraphobia*	5.00 (1.75–8.00)	4.00 (0.00–7.00)	107.00		.058	.082
*Separation anxiety*	4.00 (2.50–10.50)	4.00 (0.00–7.00)	82.50		.089	.196
*Social anxiety*	4.00 (1.00–6.00)	6.00 (0.00–8.00)	153.00		.799	.084
*Specific phobias*	4.00 (2.00–8.00)	0.50 (0.00–7.50)	63.50		.013*	.238
*Generalised anxiety*	4.00 (1.50–8.00)	4.00 (0.25–7.75)	138.50		.330	.034
**Insistence on Sameness**						
*Agoraphobia*	4.00 (.00–6.00)	3.00 (0.00–5.00)	− .509	44	.613	.152
*Separation anxiety*	4.50 (1.50–7.25)	3.00 (0.00–5.00)	196.50		.487	.139
*Social anxiety*	4.00 (0.50–6.00)	3.00 (0.00–4.50)	223.50		.382	.129
*Specific phobias*	4.00 (0.75–6.25)	2.00 (0.00–4.00)	144.00		.127	.225
*Generalised anxiety*	4.00 (1.00–7.00)	2.00 (0.00–7.75)	200.50		.165	.205

*p < .05

*Note. n* may vary due to missing data

^a^Cohen’s *d* reported for *t*-tests, *r* for Mann-Whitney *U* tests, Cramer’s *V* for chi-square tests

## Discussion

In this study, DSM-5 and ASD-related anxiety symptomatology were examined in two genetic syndromes at high risk for anxiety. The CdLS and FXS groups were comparable for chronological age, level of adaptive functioning and ASD symptomatology. Associations between presence of anxiety symptomatology with chronological age, adaptive functioning, ASD characteristics and repetitive behaviour were assessed.

### Prevalence and profiles of anxiety symptomatology

DSM-5 anxiety symptomatology was highly prevalent in both groups with most individuals reported to show behaviour consistent with at least one anxiety type. This is greater than previous estimates in individuals with ID, autistic individuals and CdLS and FXS specifically [1, 2, 4, 5, 8, 31] but expected as subthreshold symptomatology was assessed as opposed to symptomatology meeting threshold for diagnosis. However, consideration of subthreshold conditions is necessary as many individuals may fall into this category if they show presentations of anxiety that do not meet criteria that are based on ‘typical’ presentations or difficulties self-reporting internal states due to low cognitive and expressive language skills [19]. Prevalence of ASD-related anxiety types were also explored in verbal individuals only and found to be prevalent in both groups (CdLS = 43.8% and FXS = 35.5% meeting ASC-ASD cut-off scores).

Visual inspection of subscales revealed greater endorsement for the uncertainty subscale in comparison to other subscales for both individuals with CdLS and FXS. This indicates that, in individuals at high risk for anxiety, difficulties with Intolerance to Uncertainty may underlie presentations of symptomatology. Both presence of subthreshold DSM-5 and ASD-related anxiety is associated with decreased wellbeing and reduced quality of life [18]. However, due to poor recognition of ASD-related anxiety in clinical guidelines and without a diagnosis of a DSM-5 anxiety disorder, individuals are unlikely to have access support and so these high rates of anxiety here have critical implications for these groups.

Low endorsement of separation anxiety and performance anxiety on the ASC-ASD in comparison to the uncertainty subscale was surprising as theoretically it may have been expected that these would broadly map onto DSM-5 separation anxiety and social anxiety, which were highly reported in both groups. However, individuals reported to show separation anxiety symptomatology formed a comparatively small subgroup which may have been masked when scores were pooled and assessed at group level. Additionally, previous literature has described atypical presentations of social anxiety type behaviour in both CdLS and FXS groups, which may have confounded measurement of these when assessments developed for autistic individuals or the general population are used [4, 32]. It should also be noted that, whilst the ASC-ASD and KSADS may be described as assessing the same construct at a broad level (i.e. anxiety for social situations or separation from others), *the way* these constructs are conceptualised and thus measured may differ. That is, autistic and neurotypical individuals may evidence different profiles and presentations of symptoms for the same type of anxiety construct. Thus, the lack of agreement in measures may indicate that, whilst anxiety for social situations and separation from others are present in CdLS and FXS, this does not appear to be captured by an ASD-related presentation of such anxiety. A final consideration may be the inherent difficulties in assessing anxiety in individuals with severe to profound ID, as the ASC-ASD was not created for such individuals and is not appropriate for minimally verbal individuals [31]. The items of the questionnaire require informants to comment on individuals’ internal thoughts and feelings which is challenging for individuals with low cognitive and expressive language skills [19]. Comparatively, uncertainty subscale items focus on observable, behavioural indicators of anxiety. This may indicate why caregivers endorsed these items more, as they had more confidence in identifying these behavioural markers. These issues highlight a need for research to develop more robust assessments which are appropriate for individuals with ID and sensitive to atypical presentations of anxiety.

Cross-syndrome comparisons, controlling for adaptive behaviour and ASD phenomenology, revealed no significant group differences for ASD-related or DSM-5 anxiety except for social anxiety which was more prevalent in the FXS group. Difficulties in social situations are prominent in both CdLS and FXS behavioural phenotypes [15]. However, this finding suggests this is particularly significant for individuals with FXS, although it should be noted this was also highly reported in the CdLS group.

In general, comorbidity and co-occurrence within and between DSM-5 and ASD-related anxiety was high in both syndrome groups. This is consistent with high rates of comorbidity in the idiopathic ASD literature [3] and may indicate that presence of one anxiety type places individuals at greater risk of developing other types. Additionally, where high comorbidity and co-occurrence exists, a singular underlying construct (e.g. an ‘atypical’ presentation of anxiety), causal mechanism or common risk factors may be considered [38]. Analyses revealed uncertainty was associated with generalised anxiety (anxiety response) but not specific phobias (fear response) suggesting some DSM-5 anxiety symptomatology may emerge via an Intolerance to Uncertainty in individuals with CdLS and FXS. Further research is required to investigate whether uncertainty is associated with other DSM-5 anxiety types in these groups, that is, whether presentations of many separate DSM-5 anxiety types are underpinned by a singular anxiety construct of intolerance to uncertainty. This has significant implications for the assessment and intervention of anxiety in these groups in order to target underlying mechanisms. Specifically, assessment of anxiety symptomatology should carefully consider whether this appears indicative of an intolerance to uncertainty, with interventions such as coping with uncertainty in everyday situations [39] considered where this is the case.

Interestingly, co-occurrences between specific DSM-5 anxiety types appeared to differ across syndrome groups which may be indicative of separate underlying mechanisms. For example, a significant association was identified between occurrence of social anxiety symptomatology and selective mutism in the CdLS group only. As discussed previously, social anxiety had significantly greater prevalence in FXS; however, cross-syndrome comparisons of selective mutism indicated a greater prevalence in CdLS, approaching significance. The disproportionate rates of selective mutism in CdLS, apparent dissociation between social anxiety and selective mutism relative to FXS, alongside significant co-occurrence of these anxiety types in CdLS may be indicative of a syndrome-specific presentation. Individuals with CdLS are reported to show executive dysfunction and anecdotal evidence of difficulties initiating movement and speech [40]. Thus, in CdLS, rather than appearing downstream of social anxiety, selective mutism may contribute to the emergence of anxiety due to difficulties following and keeping the pace of conversations [7, 41]. This also has implications for how anxiety related to social situations may emerge in individuals with ID and ASD more broadly.

### Associated characteristics

Finally, associations with participant characteristics including chronological age, adaptive functioning, ASD characteristics and repetitive behaviour were investigated in these high-risk groups. No significant associations between adaptive functioning and any DSM-5 or ASD-related anxiety type was identified except for specific phobias in CdLS which were reported significantly more in individuals with lower adaptive functioning. This is consistent with previous studies identifying ID as a putative risk marker for anxiety [1]. However, the lack of other associations between adaptive functioning and anxiety is interesting. Few studies report presence of anxiety symptomatology in individuals with level of ability as low as that described here (indicated by VABS-II standard scores). This suggests that whilst anxiety is prevalent in individuals with severe to profound ID, within this population, there are other person characteristics which are more predicative of risk.

No significant associations between chronological age and any DSM-5 anxiety were identified except for specific phobias in FXS which were significantly lower in the older group. No change with age in the CdLS group is somewhat inconsistent with previous evidence where age-related changes are described [5, 8, 33]; however, some studies also show evidence of anxiety emerging in childhood and persisting over time [15]. These findings support these and suggest that anxiety is prevalent across children and adults in CdLS and FXS. The lack of trajectory for separation anxiety in the CdLS and FXS groups and specific phobias in individuals with CdLS is interesting as these are reported to be more common in children than adults in the typical population and so a downward trajectory would be expected [18, 23]. Specific phobias were less prevalent in the older FXS group, broadly following typical developing trajectories [18, 23]. Thus, the relatively heightened presence of specific phobias in adults with CdLS may be of interest.

Associations between anxiety and ASD phenomenology were not significant, except for the uncertainty subscale, such that greater anxiety was associated with greater severity of ASD characteristics. This is consistent with Intolerance for uncertainty being conceptualised as an ASD-related anxiety trait [19, 42]. Notably, the lack of significant associations between ASD characteristics and social anxiety is important as it suggests, in CdLS and FXS, anxiety symptomatology presents independently from ASD. Assessment and recognition of social anxiety in individuals with ID is often confounded by an ASD presentation and limited by diagnostic overshadowing. This therefore has critical implications for clinical practice and the delivery of appropriate interventions, that is, whether interventions for difficulties in social situations draw from those recommended for autistic individuals or for social anxiety.

Associations were observed between specific repetitive behaviours in the CdLS group and agoraphobia, separation anxiety, specific phobias and uncertainty. Taken together, these indicate that individuals with greater propensity to show repetitive behaviours are more likely to show anxiety. Importantly, this association may not be driven by presence of ASD symptomatology, as no significant associations between DSM-5 anxiety and ASD characteristics were identified, as discussed previously. This suggests repetitive behaviour may be a behavioural marker of anxiety in this group, consistent with theories of behavioural equivalents of mental health difficulties [42]. However, as repetitive behaviours do not occur only in the context of an anxiety response, these behaviours lack specificity to be used reliably in diagnoses of anxiety unless a specific change in baseline frequency or severity is identified and so may have utility as supplementary information. Finally, the breadth and variability of repetitive behaviours implicated (as shown in Table 7) could be indicative of distinct underlying mechanisms for anxiety types. Specifically, insistence on sameness has been highlighted as a possible coping mechanism for autistic individuals to reduce demand in anxiety provoking situations [23]. In the typically developing literature, preference for sameness and routine is observed in young children and is proposed to serve as an adaptive regulation strategy before being replaced by more sophisticated strategies as the child grows older [43]. Therefore, the association between uncertainty and insistence on sameness here indicates preference for routine may also serve as an anxiety regulation strategy for individuals with CdLS, specifically for anxiety emerging from an intolerance to uncertainty.

## Limitations and conclusions

This is the first study to examine profiles of anxiety symptomatology across individuals with CdLS and FXS. The two groups were comparable for presence of ASD characteristics meaning anxiety symptomatology could be assessed independently of ASD, which may present similarly to anxiety [19]. Additionally, the sample size included here is relatively high compared to other studies of genetic syndromes and so represents a significant strength. However, for analyses examining associated characteristics, the groups were divided and the resulting subgroups may have been too small to detect significant change. Thus, analyses may have been underpowered and so results should be interpreted with caution. Furthermore, due to small sample size and to limit number of comparisons, no analyses could be conducted between the least common anxiety types with chronological age and ASD characteristics, and between anxiety types with repetitive behaviour for the FXS group, which is a significant limitation. Additionally, the bands selected for the chronological age, adaptive behaviour and ASD characteristics analyses meant a large range of ages and scores were clustered together, which may have led to subtle nuances in trajectories and level of ability and ASD profiles with anxiety being missed. Finally, a strength of this study was the CdLS and FXS groups were comparable on key person characteristics including chronological age, level of adaptive functioning and ASD symptomatology which meant syndrome specific profiles could be investigated.

In summary, this study indicates that in individuals with CdLS and FXS both DSM-5 and ASD-related anxiety symptomatology are highly prevalent in both children and adults. In the CdLS group, repetitive behaviour was shown to be associated with specific types of anxiety which may inform diagnoses of these. Finally, the dissociable relationship between uncertainty and generalised anxiety and uncertainty and specific phobias suggests there may be multiple underlying mechanisms for anxiety types in these syndrome groups. This has important implications for clinical practice particularly in the selection of appropriate interventions for individuals with ID and ASD which specifically target these mechanisms.

## Data Availability

Not applicable.

## Abbreviations

ABC: Adaptive Behavior Composite
ASC-ASD: Anxiety Scale for Children-Autism Spectrum Disorder
ASD: Autism spectrum disorder
CdLS: Cornelia de Lange syndrome
DSM-5: Diagnostic and Statistical Manual of Mental Disorders, 5th Edition
FXS: Fragile X syndrome
ID: Intellectual disability
K-SADS: Kiddie-Schedule of Affective Disorders
RBQ: Repetitive Behaviour Questionnaire
SRS-2: Social Responsiveness Scale-Second Edition
VABS-II: Vineland Adaptive Behavior Scale-Second Edition

## Footnotes

1 Minimally verbal participants identified by caregiver report of individual speaking few or no words.
